# Real-Time Stereotactic MRI-Guided Sclerotherapy with Bleomycin-Polidocanol Foam: Illuminating Inaccessible Venous Malformations

**DOI:** 10.3390/jcm14217509

**Published:** 2025-10-23

**Authors:** Xuan Jiang, Zimin Zhang, Li Hu, Hongyuan Liu, Jingwei Zhou, Hui Chen, Xi Yang, Xiaoxi Lin

**Affiliations:** 1 Department of Plastic and Reconstructive Surgery, Shanghai Ninth People’s Hospital, Shanghai Jiao Tong University School of Medicine, 639 Zhizaoju Road, Huangpu District, Shanghai 200011, China; jiangx0110@163.com (X.J.); huli89@foxmail.com (L.H.); brooks_ryo@163.com (H.L.); zhjngw@gmail.com (J.Z.); chenhui9801640@163.com (H.C.); 2 Department of Radiology, Shanghai Ninth People’s Hospital, Shanghai Jiao Tong University School of Medicine, 639 Zhizaoju Road, Huangpu District, Shanghai 200011, China; drzhangziminsh@163.com; 3 Department of Laser and Aesthetic Medicine, Shanghai Ninth People’s Hospital, Shanghai Jiao Tong University School of Medicine, 639 Zhizaoju Road, Huangpu District, Shanghai 200011, China

**Keywords:** venous malformation, real-time MRI, sclerotherapy, bleomycin-polidocanol foam, RSMS-BPF

## Abstract

**Objectives:** Venous malformations (VMs) that infiltrate the muscular layer, involve or are closely adjacent to critical nerves or vessels, or are located deep within or very close to major organs in the thoracic or abdominal cavities are challenging to access during sclerotherapy, which we defined as inaccessible VMs. This study proposed an integrated real-time stereotactic MRI-guided sclerotherapy with bleomycin-polidocanol foam (RSMS-BPF) for the treatment of inaccessible VMs, focusing on its clinical feasibility, efficacy, and safety. **Methods:** A retrospective study was conducted involving patients treated with RSMS-BPF between 2019 and 2021. During the sclerotherapy, the intraoperative magnetic resonance imaging (MRI) was combined with an optical navigation system to guide precise needle placement and track BPF, a foam sclerosant optimized for MRI visibility. Radiological response was assessed by lesion volume, while clinical improvement was evaluated through patients’ description of their symptoms. Rigorous follow-up and documentation of complications were conducted. **Results:** A total of 42 patients (mean age 23.6 ± 1.6 years; 18 males) were treated in 64 sclerotherapy sessions. The treatment achieved an overall response rate of 89.5%. Imaging analysis revealed an average lesion volume reduction of 59.6%. 57.9% of patients achieved good or excellent radiological responses. After a median follow-up of 12.25 months, 60.53% of patients reported complete or significant relief. Lesion depth did not affect treatment efficacy (*p* = 0.43). Minor complications included skin hyperpigmentation (5.3%, 2/38) and blisters (2.6%, 1/38), with no major complications observed. **Conclusions:** RSMS-BPF demonstrated satisfactory efficacy and safety in VMs treatment, particularly for inaccessible VM lesions. It enables authentic real-time dynamic tracking during sclerotherapy, achieving unparalleled precision targeting while minimizing procedural risks. These findings strongly support routine integration of RSMS-BPF as first-line therapy for complex vascular malformations with critical anatomical constraints.

## 1. Introduction

Vascular malformations originate from embryogenic aberrations in vasculogenesis, resulting in the persistence of abnormally structured vessels, including capillary, lymphatic, venous, arteriovenous, and mixed malformations. Among these, VM represents one of the most common subtypes, approximately 70% of all vascular malformations [1]. VMs are characterized by abnormally dilated, thin-walled venous channels that can infiltrate surrounding soft tissues and occasionally involve muscle or bone. Clinically, VMs may cause pain, swelling, functional impairment, and aesthetic concerns, and they often present therapeutic challenges due to their infiltrative nature and variability in size and location [1].

Sclerotherapy involves percutaneous injection of a sclerosing agent directly into the lesion to induce endothelial damage, thrombosis, and subsequent fibrosis, ultimately reducing the size and symptoms of the malformation [2]. Various sclerosants, such as ethanol, polidocanol, and bleomycin, have been employed, each with specific efficacy and safety profiles [2]. As the first-line treatment of VMs, traditional sclerotherapy has proven effective for superficial and moderately sized lesions, and has been estimated as a more effective and safer alternative than surgical resection, especially in patients with diffuse and multifocal lesions [2].

Nevertheless, for some extensive lesions that are challenging to access during sclerotherapy, with indistinct boundaries and those adjacent to vital nerves or blood vessels, or those which are deeply located or terribly close to some major organs in chest and abdomen, the issue of how to achieve a more precise and safer treatment has been under discussion among doctors [3]. Intra-procedural guidance commonly relies on ultrasound (US), computed tomography (CT), and X-ray fluoroscopy, though each has its limitation [4]. MRI is a gold standard for evaluating venous malformations, due to its superior soft tissue contrast, high spatial resolution, and ability to delineate lesion extent and involvement of surrounding structures noninvasively. It addresses key limitations of conventional imaging in VM management by providing unparalleled soft-tissue contrast and panoramic anatomical visualization, enabling precise delineation of lesion boundaries from critical adjacent structures. Furthermore, its radiation-free nature permits serial imaging surveillance and repeat interventions, which is particularly critical for pediatric VM patients requiring staged therapies [5].

However, traditional MRI-guided navigation is time-consuming, and may not facilitate real-time observation of needle puncture and accurate adjustment of the surgical approach during surgery, potentially impacting the therapeutic process [6]. Intraoperative MRI enables the evaluation of the extent of resection during treatment through intraoperative visualization of the lesion, thereby enhancing the precision of the therapy, which is a highly promising therapeutic approach. However, it’s known to burden the surgical workflow due to the long acquisition time [7]. In this research, we introduced an MRI-based intraoperative imaging and navigation system and an optical stereotactic module prototype for sclerotherapy in VM patients, with better resolution, faster image acquisition and a smaller physical footprint thus facilitating its integration into clinical practice [8]. We evaluated the feasibility and efficacy treating deep-located VMs via this system, and concluded that the combination of intraoperative MRI and optical navigation can facilitate real-time imaging of puncture process, which is integral for safer treatment of deep lesions.

## 2. Materials and Methods

### 2.1. Research Objects Selection

This study was approved by the Institutional Review Boards of our center (IRB Nos. SH9H-2024-T397-1). In this retrospective cohort study, we analyzed the cases and imaging data of 42 patients who underwent sclerotherapy in our center between 2019 and 2021. The participant group consisted of 42 individuals (18 male, 24 female), with a mean age of 23.6 years and an age range spanning from 8 to 56 years (Table 1).

**Figure 1:**
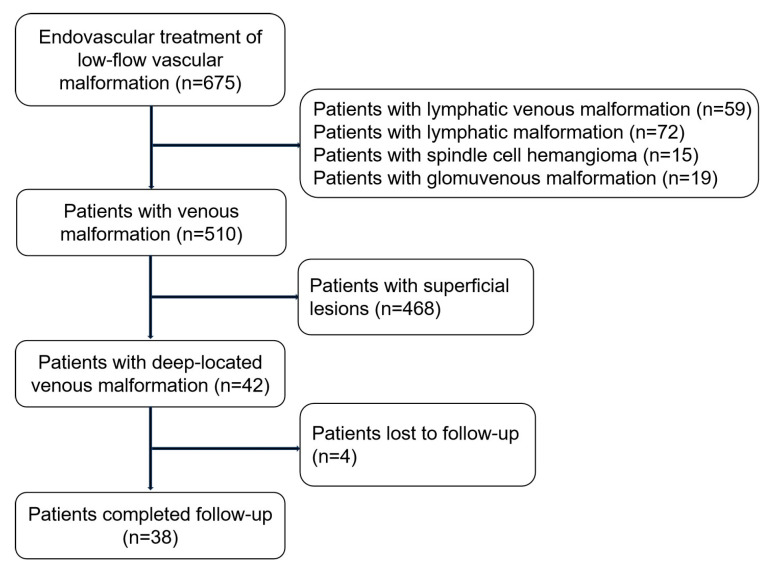
Flowchart of the inclusion and exclusion criteria of the patients. Exclusion criteria include other types of low-flow vascular malformations, superficial lesions. n = number of patients.

Patients with deep-located VMs were included (Figure 1). Deep-located VMs were defined as lesions demonstrating invasion or infiltration of the muscular layer, or as lesions located more than 3 cm beneath the skin surface. This cohort specifically analyzed patients who met these criteria. Preoperative coagulation profiles were assessed, including prothrombin time, a full blood count, D-dimer, activated partial thromboplastin time, and fibrinogen levels. Informed consent and ethics committee approval were obtained.

### 2.2. Real-Time Intraoperative MRI and Optical Navigation Systems

**Figure 2:**
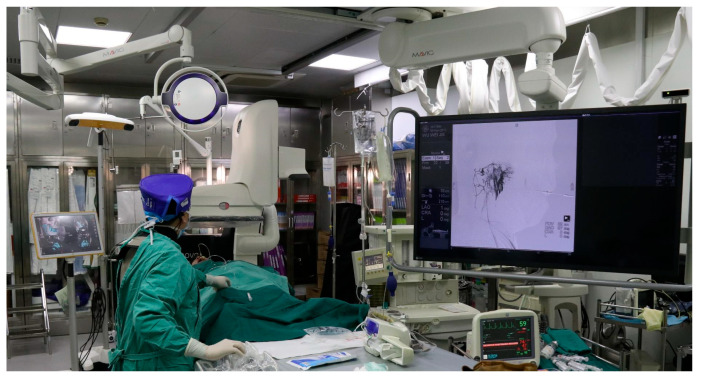
Intraoperative process of real-time MRI combined with optical navigation system. In addition to the MRI scanner, the setup includes a detachable bedside positioning frame that can be mounted at variable locations along the examination table to accommodate different treatment sites; an infrared tracking camera that captures reflected light from trackable fiducial markers to compute the real-time spatial relationship among instruments, the patient table, and the MR scanner; and two models of MR-compatible puncture needles provided to suit different lesion depths and access trajectories.

The navigation system consists of a movable 3D infrared camera (Polaris Spectra^®^, NDI, Waterloo, ON, Canada) and optical markers attached to the MRI scanner, patient table, and needle holder (Figure 2).

**Figure 3:**
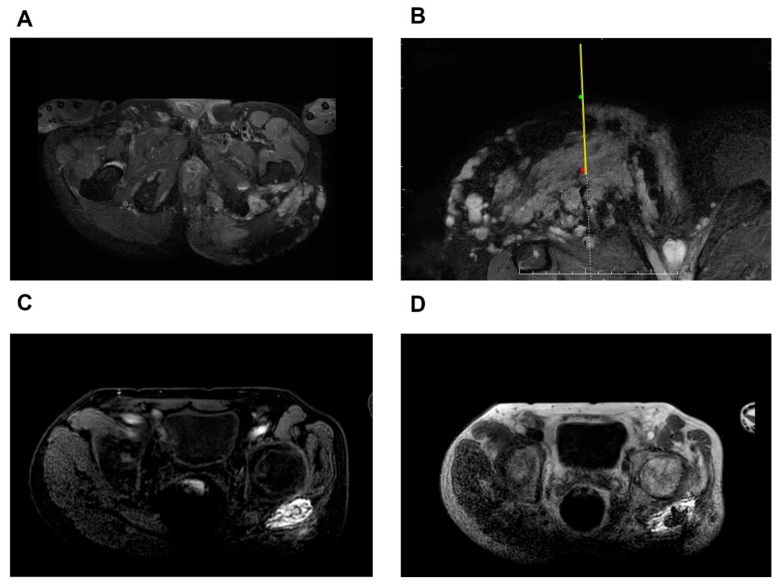
Diagram of the surgical process. The green spots indicate the entry point, the red dots represent the puncture target, the yellow lines illustrate the needle, and the dotted lines depict its trajectory toward the target. (**A**) Pre-treatment axial fast mDIXON T2-weighted image demonstrating the venous malformation located in the left hip joint; (**B**) fast mDIXON T2-weighted image showing the position of the needle and its tip reaching the target area; (**C**) FFE T1-weighted image following the injection of contrast agent; (**D**) fast mDIXON T2-weighted image post-injection of the sclerosing agent. FFE, fast field echo; mDIXON, modified DIXON.

Using an optical tracking system (Northern Digital Inc., Waterloo, ON, Canada), the needle trajectory to the target was preplanned. Navigation guidance was then achieved using a surgical navigation system (Vector Vision, Brain-LAB, München, Germany) equipped with the iPlan planning software platform (Brain-LAB, München, Germany) (Figure 3). All interventions were conducted in a 3.0 T fully open MRI scanner with a horizontal magnet gap (EMT-4000F, EMT, Shanghai, China). The MRI system was equipped with an additional workstation, a flat-screen monitor, and a wireless mouse and keyboard for sequence initiation and navigation control within the MRI room. A flexible single-loop surface coil (EMT, Shanghai, China) with a 30/40 cm inner diameter was used for all procedures. MR imaging data were acquired as T1-weighted, FFE (fast field echo) sequence (TR, 3.2 ms; TE, 2.0 ms; slice thickness, 3D; field of view, 268 × 236; voxel size, 1.49 × 1.49 × 6.00 mm; Philips Healthcare, Best, The Netherlands), and T2-weighted, mDIXON (modified DIXON) sequence without a gap (TR, 3000 ms; TE, 75 ms; slice thickness, 3 mm; field of view, 352 × 375; voxel size, 0.85 × 1.01 × 4.00 mm; Philips Healthcare) (Supplementary Table S1).

### 2.3. Sclerosant Foam

BPF was prepared by dissolving bleomycin powder (15 mg, 15,000 IU; Nippon Kayaku, Tokyo, Japan) in 4 mL of 3% polidocanol (Aethoxysklerol; Kreussler Pharma, Wiesbaden, Germany). Sclerosant foam was generated via the Tessari technique using two 5 mL syringes connected by a 3-way stopcock at a 4:1 air-to-liquid ratio. The mixture was emulsified through 20 cycles of rapid syringe agitation. The prepared foam syringe was immediately detached and stored vertically (plunger down) to stabilize foam architecture.

### 2.4. Procedure

Before the operation, all patients were trained to breathe slowly and steadily. Subsequently, each patient was placed on the MRI table in prone position, with the flexible single-loop surface coil securely affixed at the designated location.

Axial, coronal, and sagittal FFE T1-weighted and mDIXON T2-weighted images were obtained for anatomical assessment of the target region and to delineate adjacent critical structures. Subsequently, the acquired images were then transferred to the navigation system’s workstation, where the procedural pathway, puncture site, and target coordinates were defined on the pre-acquired scans. In essence, the position, orientation, and trajectory of the coaxial needle are monitored in real-time within a multiplanar, virtual MRI environment displayed on the monitor. Following motion calibration, the patient table was repositioned to clear the aiming imaging field.

All procedures were conducted under local anesthesia, utilizing 2% lidocaine. Subsequently, an 18G MR-compatible coaxial needle was meticulously advanced towards the target under the guidance of a real-time virtual navigation system, facilitated by an optical tracking system. Upon reaching the target along the pre-planned trajectory, the patient table was repositioned to align with the initial MR imaging setup. A repeat mDIXON T2-weighted sequence was then executed to confirm the needle’s puncture location.

**Figure 4:**
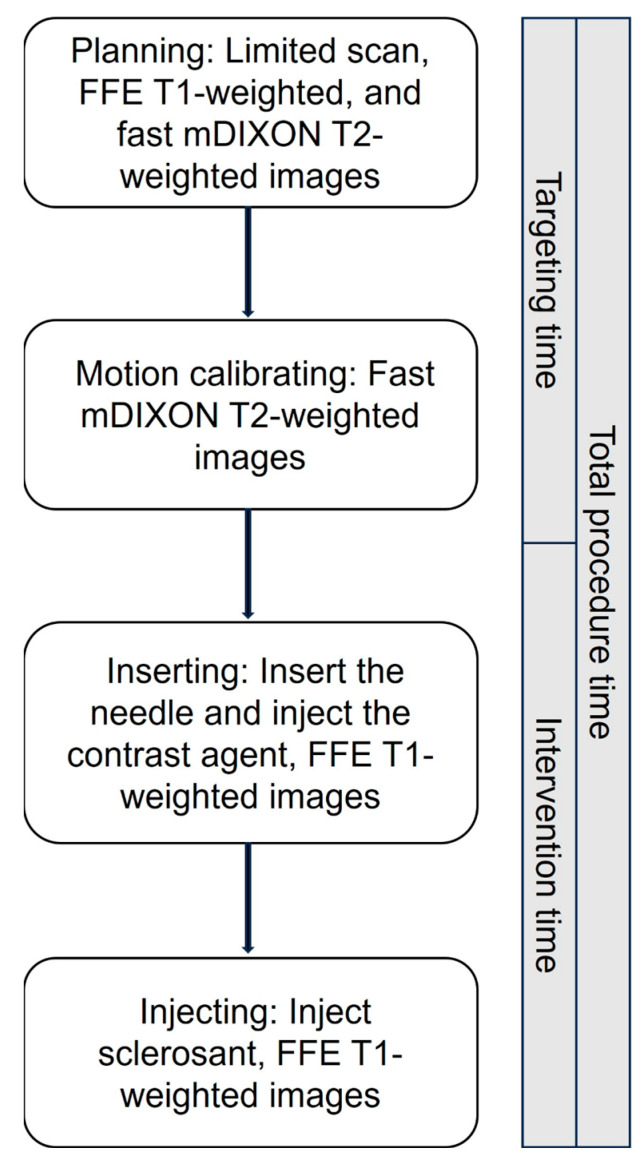
Schematic of procedure workflow for the real-time intraoperative MRI and optical navigation system-guided sclerotherapy treating VM. Axial, coronal, and sagittal FFE T1-weighted and fast mDIXON T2-weighted images were acquired to assess the anatomy of the target area and identify surrounding critical structures. These images were then transmitted to the navigation system workstation. Using the pre-scan images, the surgical path, entry point, and target area were carefully planned. The puncture needle was advanced to the lesion site under the navigation system’s guidance. Once the needle was positioned, a contrast agent was injected, and FFET1-weighted images were obtained to confirm the injection site. Subsequently, the sclerosant was injected, and additional FFET1-weighted images were acquired to verify the sclerosant’s location. FFE, fast field echo; mDIXON, modified DIXON.

At each session, the number of injection sites is determined by the lesion size. Contrast agent administration was guided by real-time phlebography and FFE T1-weighted imaging (T1WI) to verify needle placement, lesion boundaries, and venous drainage. A maximum of 20 mL BPF was administered per procedure, with immediate imaging confirmation (Figure 4).

Vital signs were continuously monitored without tourniquet use until sclerosing foam achieved complete lesion filling under visual guidance.

### 2.5. Outcome Evaluation

Several sociodemographic and clinical variables: sex, age, preoperative symptoms, anatomical localization, lesion depth, sessions, preoperative and postoperative lesion volumes, follow-up time, clinical improvement, and postoperative complications. Lesion depth was accessed by measuring the shortest straight-line distance from the lesion center to the body surface. Focal lesions are radiologically defined by well-defined borders, homogeneous signal intensity, and absence of admixed normal tissue, whereas diffuse lesions exhibit poorly defined margins, heterogeneous signal characteristics, and infiltrative interdigitation with surrounding parenchyma under MRI.

Lesion volumes were modeled as ellipsoids using the formula 0.523 × abc (a = anteroposterior, b = transverse, c = cranio-caudal axes).

Radiological response was graded by the calculated volume reduction: 1 = unchanged or minor response (<25% decrease), 2 = medium response (≥25% decrease up to <50% decrease), 3 = good response (≥50% decrease up to <75% decrease) or 4 = excellent response (≥75% decrease). Clinical improvement was assessed based on patient subjective description by the following scale: 1 = minimal improvement; 2 = good improvement; 3 = significant improvement; 4 = complete relief.

### 2.6. Statistical Analysis

Statistical analyses were performed with SPSS 26.0. Normality of continuous variables was tested via Kolmogorov–Smirnov; normally distributed data were reported as mean ± SD, non-normal data as median. Group comparisons used t-tests (normal) or Mann–Whitney U tests (non-normal), with statistical significance set at *p* < 0.05 (two-tailed).

## 3. Results

### 3.1. Patient Characteristics

The study included 42 participants (18 male, 24 female). The average age was 23.6 ± 1.6 years old, ranging from 8 to 56. Among them, 16 patients were under 18 years old, while 26 were over 18. All patients (42/42) reported swelling as a primary symptom, with 83.3%patients (35/42) also experiencing pain. Additionally, 14.29% of patients (6/42) experienced limb dysfunction. Preoperative D-dimer and fibrinogen levels showed no focal coagulopathy.

Within this cohort, the lesions were distributed across the head and neck region (n = 4), upper extremities (n = 6), lower extremities (n = 22), and trunk (n = 10). Focal lesions were identified in 35.7% of patients (15/42), whereas 64.3% of patients (27/42) had diffuse lesions. These inaccessible lesions have a median depth of 4.78 cm, while diffuse lesions are deeper than focal ones (*p* = 0.045).

All 42 patients successfully underwent sclerotherapy, with a total of 64 procedures performed (median = 1.3). The most extensively treated patient underwent six sessions. 56 of 64 procedures targeted multiple lesions or components, treating 86 sites.

Postoperative follow-up was conducted according to our clinical standards, with a median duration of 12.3 months (range: 3–96 months). Four patients were lost to follow-up after treatment. Longer follow-up periods depended on patient prognosis (Table 1).

### 3.2. VM Volumes

We performed a volume calculation based on ellipsoid volume formula to assess the lesion volumes of the patients. The average time from surgery to the final MRI follow-up was documented. The median volume of the lesions before intervention was 181.37 mL, while the median volume post-intervention was 68.34 mL, with a median reduction of 78.74 mL. There was a significant decrease in lesion volume after treatment, with an average reduction of 59.6% (*p* = 0.011). Notably, 29.0% of patients (11/38) exhibited excellent response, 29.0% of patients (11/38) showed good response, 34.2% of patients (13/38) showed medium response, and 7.9% (3/38) had only minor response. Furthermore, no correlation was observed between lesion depth and the extent of volume reduction (*p* = 0.425) (Table 2).

**Figure 5:**
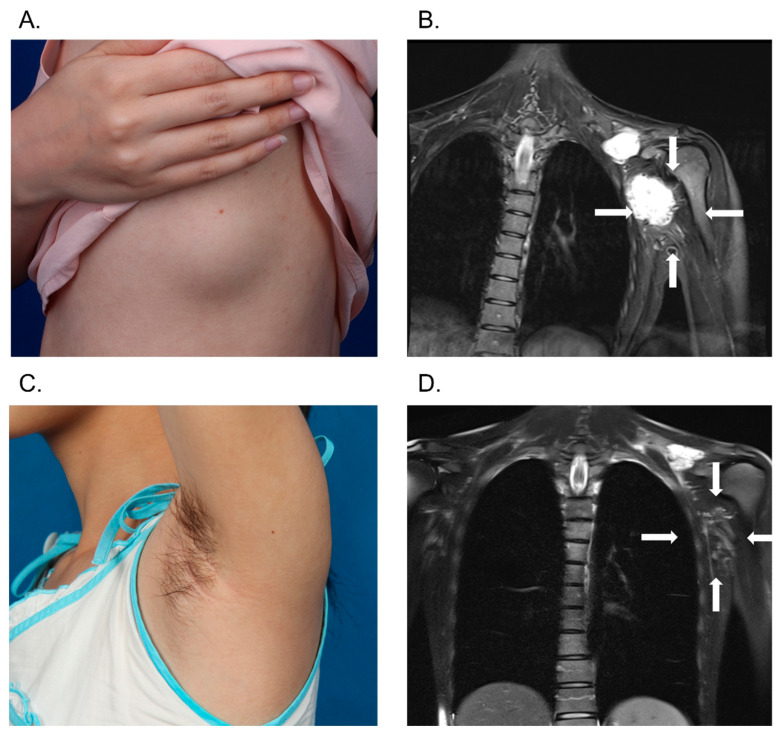
Illustration of a focal VM case. A 21-year-old female suffered from pain and swelling, cased by focal VMs in the left supraclavicular and axillary regions (**A**). Pretreatment T2-weighted MRI revealed a hyperintense mass in the left shoulder region, anatomically demarcated by white arrows (**B**). The patient underwent real-time MRI-guided sclerotherapy using BPF. Subsequent two-year follow-up reported significant clinical relief (**C**), and MRI demonstrated volume reduction of the VM lesions (**D**). VM, venous malformation; MRI, magnetic resonance imaging; BPF, bleomycin-polidocanol foam.

**Figure 6:**
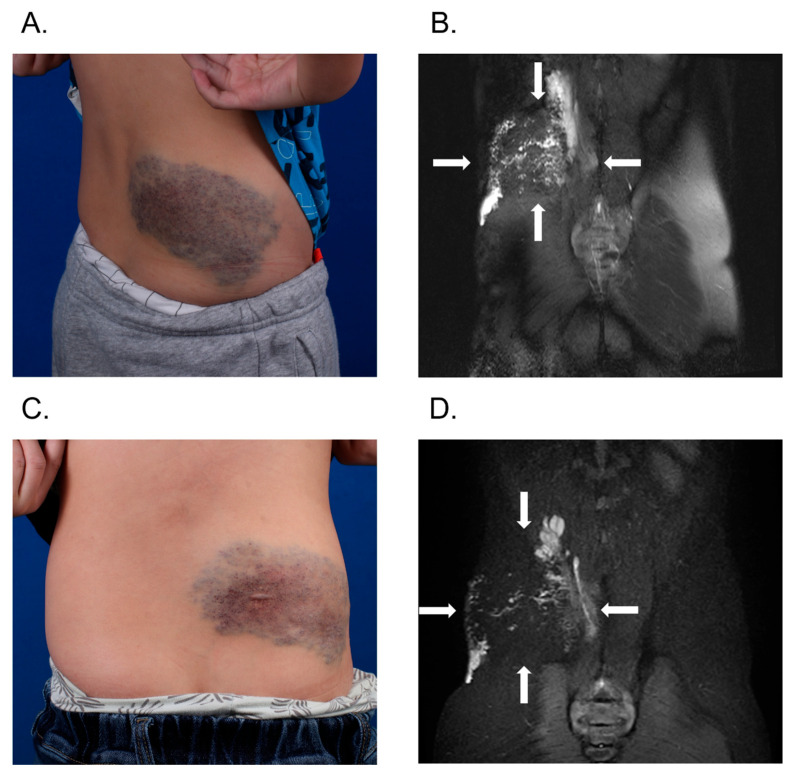
Illustration of a diffuse VM case. An 8-year-old male patient had diffuse VM in the right back. Before treatment, clinical appearance showed extensive mass on the right back (**A**), and T2-weighted MRI showed hyperintense mass of the right back (**B**). After one session of real-time MRI-guided BPF sclerotherapy, significant improvement of VM lesions was obtained (**C**). After 2 years of follow-up, T2-weighted MRI showed that the VM lesions were significantly smaller (**D**). VM, venous malformation; MRI, magnetic resonance imaging; BPF, bleomycin-polidocanol foam.

For focal lesions, the average volume reduction per patient was 71.9%, significantly higher than the 53.1% observed in diffuse lesions (*p* = 0.016). Clinical outcomes for focal and diffuse lesions are presented in Figure 5 and Figure 6, respectively. Our analysis revealed significantly superior therapeutic outcomes in patients with focal VMs compared to those with diffuse lesions (*p* = 0.019). Among patients with focal lesions, 84.6% (11/13) showed good or excellent improvement. In contrast, all 3 patients with minor improvements had diffuse lesions. Only 44% (11/25) of patients with diffuse lesions experienced satisfactory improvement (Table 3).

### 3.3. Symptom Improvement

The overall response rate attached 89.5% in this treatment. After treatment, 97.4% of patients (37/38) showed significant relief from swelling compared to before, while 91.4% of patients (32/35) with pain and 100% of patients (6/6) with limb disfunction experienced relief.

In follow-up assessments, 60.5% of patients (23/38) reported significant or complete relief, while 39.5% experienced minor or no improvement (Table 2). Compared to patients with diffuse lesions, those with focal lesions reported significantly better postoperative improvement (*p* = 0.011). Among patients with focal lesions, 84.6% (11/13) experienced significant or complete relief. In contrast, only 48% (12/25) of patients with diffuse lesions reported satisfactory improvement. No association was found between lesion depth and patient improvement (*p* = 0.113) (Table 3).

### 3.4. Complications

Complications included skin hyperpigmentation (5.3%, 2/38) and blisters (2.6%, 1/38). These adverse effects were self-limited and minor. All adverse effects were mild and self-resolving. No severe complications were recorded, like cutaneous necrosis, systemic anaphylaxis, cerebrovascular accidents or thromboembolic events (deep vein thrombosis or pulmonary embolism). All patients were discharged within 24 h post-procedure following observation, in accordance with standard outpatient protocols.

## 4. Discussion

This single-center retrospective analysis assessed the safety and efficacy of RSMS-BPF for inaccessible VMs.

For VM requiring intervention due to hemorrhage, pain, mass effect, or cosmetic concerns, pretreatment diagnostic angiography is mandatory to verify precise intralesional needle placement prior to sclerosant administration. This prevents extravasation into perilesional tissues or even deep venous system, decreasing the risks of severe complications including cutaneous necrosis and thromboembolic events [9]. Precision is therefore essential. This information is traditionally obtained through DSA [10]. Vollherbst et al. observed 88.9% of VM patients experienced minor or major improvement through sclerotherapy under DSA image guidance [11].

However, deep located lesions, such as VMs embedded in muscle tissue, adjacent to bones or vital neurovascular structures, proposing substantial challenges for conventional image-guided treatment. Techniques like ultrasound and DSA are limited by the depth and complexity of these lesions [12]. Ultrasound, although effective for superficial structures, has significant signal attenuation in deeper tissues, particularly when bone is related. The acoustic shadowing caused by adjacent bony structures can obscure the lesion, making accurate visualization, needle localization, and placement more difficult. Consequently, it reduces the precision of the procedure and adds the risk of complications due to incorrect targeting. DSA is useful for tracking the advancement of needles and monitoring the dispersion of contrast agents, but lacks the soft tissue resolution which is necessary to distinguish VM lesions from surrounding anatomical structures, like muscles and nerves. It increases the risk of injury to these structures during intervention and affects the treatment efficacy, while increasing the radiation exposure [13].

MRI offers a superior approach for deep-located lesions which can’t be adequately characterized with US. MRI’s high-resolution and superior soft-tissue contrast enable precise differentiation of VMs from adjacent structures, even in anatomically complex regions. It’s beneficial especially in procedures like sclerotherapy, in which accurate localization of the lesion and precise delivery of sclerosants are vital [14]. The advanced MRI sequences offer not only exceptional spatial resolution but also sufficient temporal resolution to guide in real time [15]. Mara et al. reported that the average targeting time and the average intervention time decreased in MR-guided sclerotherapy, with notable symptoms improvement (82.0%) [16]. Unlike fluoroscopy, MRI-guided interventions diminish radiation exposure, which is an important consideration for pediatric patients and those requiring multiple procedures [17]. Furthermore, MRI-guided treatments use minimal amounts of diluted contrast agents, in contrast to the larger volumes of iodine-based contrast required for fluoroscopy, reducing the risk of adverse reactions [18].

Nevertheless, problems come along as well. Traditional intraoperative MRI navigation often has long acquisition window, extending the treatment time. It can elevate the risk of complications, like infection or prolonged anesthesia, and can lead to frequent scheduling challenges. Many intraoperative MRI systems do not provide real-time imaging. This limitation can hinder intraoperative decision-making, especially in dynamic situations where rapid changes in anatomy or pathology may occur, which also compromise the quality of MR image and lead to unclear or misleading images, increasing the risk of misinterpretation and potentially resulting in suboptimal surgical outcomes [19].

RSMS-BPF overcomes these shortcomings. Under optical navigation module, we are allowed to not only define optimal needle entry position and needle path, but also observe real-time puncture process and adjust our surgery plan according to the patients’ state. During the treatment, the T1-weighted rapid sequence (15–30 s) enables rapid acquisition of high-quality volumetric images, effectively combining the temporal efficiency of DSA with the spatial fidelity of MRI, while overcoming each technique’s standalone limitations. Therefore, it offers apparent advantages over traditional imaging techniques for the treatment of deep VMs. It provides unparalleled visualization, enhances procedural precision, and reduces risks associated with radiation and contrast agents, making it a safer and more effective option, especially in anatomically challenging cases [20]. Safe and precise percutaneous delivery of sclerosants is critical, and MRI’s ability to provide real-time feedback significantly reduces the likelihood of complications, such as extravasation into surrounding tissues or unintentional sclerosant flow into draining vein [14].

In our cohort, 57.9% of patients (22/38) achieved excellent or good radiological responses. All patients experienced an average lesion volume reduction of 59.6%, with significant postoperative decreases observed. Follow-up surveys indicated that 60.52% of patients reported complete or significant improvement. While this differs from the results reported by Yang et al. (84.6%) and Ni et al. (78.5%), it’s worth noting that we evaluate the outcome by calculating the volume of each lesion, and the cohort involved many patients with diffuse and extensive lesions [21,22].

We categorized patients based on lesion type into focal and diffuse groups. Our analysis revealed that patients with focal lesions had significantly better radiological responses and clinical relief compared to those with diffuse lesions. No significant correlation has been found between lesion depth and therapeutic efficacy, suggesting that our navigated sclerotherapy approach effectively mitigates the impact of lesion depth on outcomes.

Recent studies indicated that BPF enhanced sclerotherapy efficacy by improving foam stability, extending sclerosant elution, prolonging endothelial contact, and leveraging synergistic bleomycin-polidocanol action [23]. In our preliminary research, bleomycin-polidocanol foam showed better outcome in mean lesion reduction on MRI than bleomycin foam (84.6% vs. 66%), with no differences in complication rates [22]. Moreover, the foam formulation provided superior MRI visualization over liquid sclerosants, enabling precise real-time tracking during navigation.

No permanent or significant complications were observed in our cohort, with only a few minor complications including skin hyperpigmentation (5.3%, 2/38) and blisters (2.6%, 1/38). In comparison, Vollherbst et al. reported a permanent complication rate of 3.7% in their image-guided percutaneous sclerotherapy, including hemorrhage in 2 cases (7.4%), temporary sinus bradycardia in 1 case (3.7%), apart from minor complications like swelling (88.9%) and pain (63.0%) under DSA [11]. Likewise, Chen et al. recorded swelling in 11 cases (100%) [24]. Mara et al. recorded urinary retention for 24 h in 1 case (3%) in their treatments via MRI navigation [16].

The limitations of our study still exist for its retrospective, single-center design, and the absence of a comparison group treated with standard interventional therapies for deep-seated lesions. Second, the sample size was relatively small with only 43 patients included in this study. Nevertheless, the findings can still be considered valid within the background.

## 5. Conclusions

RSMS-BPF proves effective and safe for inaccessible VMs, achieving excellent lesion reduction and symptom improvement with low complication rates. Multicenter trials are required to optimize treatment protocols and assess long-term efficacy.

## Figures and Tables

**Table 1:** Patient clinical characteristics.

Characteristics	No. Patients (Percentage)
Sex	
Male	18 (42.9%)
Female	24 (57.1%)
Age (mean ± SD, years)	23.55 ± 1.64
<18	16 (38.1%)
≥18	26 (61.9%)
Anatomical localization	
Head and neck	4 (9.5%)
Upper extremities	6 (14.3%)
Lower extremities	22 (52.4%)
Trunk	10 (23.8%)
Lesion type	
Focal lesions	15 (35.7)
Diffuse lesions	27 (64.3)
Primary symptoms	
Pain	35 (83.3%)
Swelling	42 (100%)
Limb disfunction	6 (14.3%)
No. of sessions	
1	30 (71.4%)
≥1	12 (28.6%)
Follow up (median, month)	12.3

**Table 2:** Radiological and clinical outcome.

Characteristics	All VMs
Preoperative lesion volume (median, mL)	181.4
Postoperative lesion volume (median, mL)	68.3
Lesion volume reduction (median, mL)	78.7
Percentage of lesion volume reduction	59.6%
Radiological response	
Minor response (<25%)	7.9%, 3/38
Medium (>25%, <50%)	34.2%, 13/38
Good response (>50%, <75%)	29.0%, 11/38
Excellent response (>75%)	29.0%, 11/38
Symptoms relief	
Swelling	97.4%, 37/38
Pain	91.4%, 32/35
Limbs disfunction	100%, 6/6
Clinical improvement	
Minimal improvement	10.5%, 4/38
Good improvement	29.0%, 11/38
Significant improvement	34.2%, 13/38
Complete relief	26.3%, 10/38
Complications	
Skin hyperpigmentation	5.3%, 2/38
Blisters	2.6%, 1/38

**Table 3:** Comparison of focal and diffuse VMs.

Characteristics	Focal VMs	Diffuse VMs	*p* Value
Lesion depth (median, cm)	4.7	5.2	0.045
Percentage of lesion volume reduction (mean)	71.9%	53.1%	0.011
Radiological response			0.019
Minor response (<25%)	0, 0/13	12.0%, 3/25	
Medium (>25%, <50%)	15.4%, 2/13	44.0%, 11/25	
Good response (>50%, <75%)	38.5%, 5/13	24.0%, 6/25	
Excellent response (>75%)	46.2%, 6/13	20.0%, 5/25	
Clinical improvement			0.011
Minimal improvement	0, 0/13	16.0%, 4/25	
Good improvement	15.4%, 2/13	36.0%, 9/25	
Significant improvement	38.5%, 5/13	32.0%, 8/25	
Complete relief	46.2%, 6/13	16.0%, 4/25	

## Data Availability

Our data are available from the corresponding author upon request.

## Supplementary Materials

The following supporting information can be downloaded at: https://www.mdpi.com/article/10.3390/jcm14217509/s1, Table S1: Image parameter in the 3.0 T magnetic resonance imaging.
