# Supplementary material for: Real-Time Stereotactic MRI-Guided Sclerotherapy with Bleomycin-Polidocanol Foam: Illuminating Inaccessible Venous Malformations
Source: J Clin Med. 2025 Oct 23;14(21):7509. doi: 10.3390/jcm14217509 (PMC12608222; doi:10.3390/jcm14217509)
Supplement: Supplementary file 1 [file jcm-14-07509-s001.zip › jcm-3847166-supplementary.pdf]

Supplementary Table S1 Image parameter in the 3.0 T magnetic resonance imaging.

Sequences	Time of repetition (ms)	Time of echo (ms)	Matrix scan	Axial coronal and sagittal slice thickness (mm)	Axial coronal and sagittal slice gap (mm)	Total scan duration
T1-weighted FFE	3.2	1.15	268×236	3D	0.2	00:10.8
T2-weighted mDIXON	3000	75	352×375	3	0.4	02:30.0
